# AI empowered metasurfaces

**DOI:** 10.1038/s41377-020-0332-x

**Published:** 2020-05-28

**Authors:** Shuang Zhang

**Affiliations:** School of Physics and Astronomy, University of Birmingham, Birmingham, UK

**Keywords:** Optical materials and structures, Optical techniques

## Abstract

Dynamic metasurfaces are endowed with self-adaptive and learning capabilities, enabling an array of important applications ranging from satellite communications to the monitoring of human health and activities.

Metasurfaces are artificially engineered ultrathin structured interfaces that are capable of controlling the wavefront of electromagnetic waves. Due to their ultracompact nature, these interfaces are promising for many important applications ranging from imaging to information processing and communications. However, most metasurfaces demonstrated thus far are static, meaning that they have fixed functionalities once fabricated.

Thus, one of the holograms in the field of metasurfaces is to realize dynamic metasurfaces with the independent control of the scattering property of each individual unit cell, enabling arbitrary wavefront control in real time. Recently, dynamic metasurfaces have begun to receive increasing attention from the photonics and electromagnetism communities. While it remains a tremendous challenge to implement considerable dynamic tuning at optical frequencies, much progress has been made at longer wavelengths—microwave and terahertz. In particular, at microwave and radio frequencies, active electronic elements with large tunabilities exist, paving the wave towards dynamic metasurfaces with real-time modulation.

Coded metasurfaces are one of the most promising candidates for implementing dynamic tuning^1,2^. In coded metasurfaces, each subwavelength pixel incorporates an electrically or optically tunable diode^3^, which can switch between two different states with highly contrasting reflection amplitudes and phases, leading to a digitalized 2D profile that can be reconfigured in real time. These digitalized programmable metasurfaces are capable of arbitrarily controlling the wavefront of a reflected beam, and they greatly expand the functionalities that are useful for applications such as beaming, focusing or holographic movies.

In two recent papers in Light: Science and Applications, three groups from Southeast University, Peking University, and National University of Singapore, based on their recent progress in dynamic metasurfaces, took another leap forward in making smart metasurface devices for important real-world applications.

In the paper entitled “Smart metasurface with self-adaptively reprogrammable functions” by Qian Ma et al., the authors exploit reprogrammable metasurfaces to construct a self-adaptive smart system that can reconfigure itself based on the information it receives from a sensor^4^. To implement this concept, the authors combine the metasurface with a gyroscope, which can sense the orientation of the metasurface. The orientation information is then fed into a microcontroller unit (MCU), which can calculate the required phase for the metasurface to maintain the same radiation direction for the reflected wave regardless of its orientation, based on a fast inverse design algorithm, as shown in Fig. 1. This principle is experimentally implemented, which shows very good performance in terms of the directivity and overall efficiency. This new metasurface-based technique will be very promising for applications in aviation, and it could replace complicated conventional approaches.

**Fig. 1:**
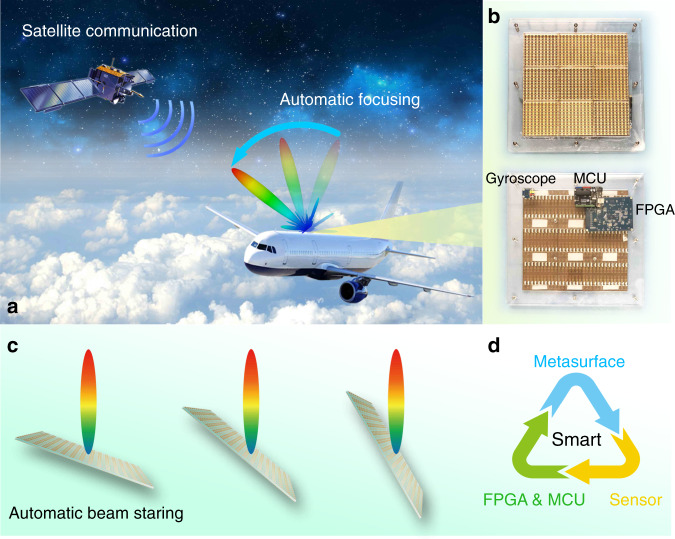
Principle of the operation of self-adaptive metasurfaces. **a** Potential application of the adaptive metasurface system in satellite communications and aviation. **b** The front and back views of the fabricated metasurface. **c** Schematic of adaptive beam steering – the tilting of the metasurface does not affect the direction of the reflected wave. **d** The key components of smart metasurfaces

In another paper entitled “Intelligent metasurface imager and recognizer” by Lianlin Li et al., the authors constructed a smart EM camera system that combines high-resolution imaging, pattern recognition, and the active control of the radiation, empowered by artificial neural networks (ANNs)^5^. In this system, the reprogrammable metasurface plays a pivotal role both as a spatial microwave modulator for image acquisition based on compressive sensing and as an adaptive focusing element to direct the microwaves to the desired spots. The neural networks function as the brain of the system, which coherently combines the functions of image processing, the identification of spots of interest, and the calculation of the phase pattern of the metasurface. As a proof of concept, the authors show that the smart metasurface system is capable of generating high-quality imaging of the human body, identifying the body parts of interest (hand and chest), monitoring both the breathing rate and recognizing hand gestures (Fig. 2). Interestingly, the EM camera can also operate in a passive mode, merely relying on the stray 2.4 GHz Wi-Fi signals that exist almost everywhere. The intelligent EM “camera” based on metasurfaces is compact and inexpensive, which holds promise for many applications for future smart cities, smart homes, human-device interactive interfaces, healthy monitoring and safety screening.

**Fig. 2:**
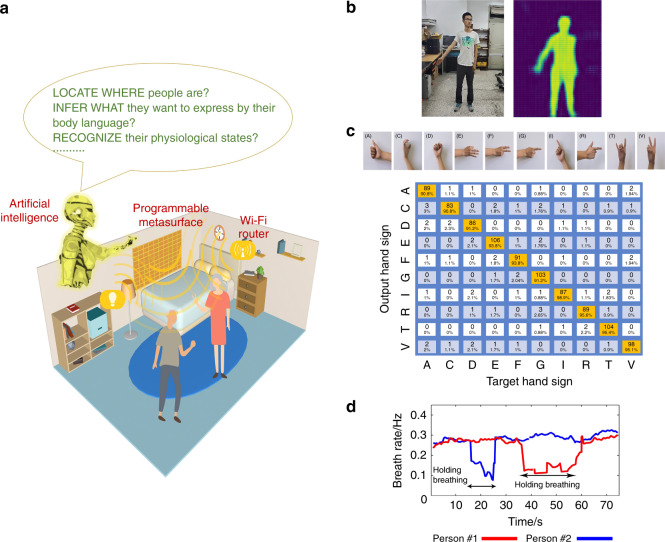
Demonstration of the imaging and recognition of human activities. **a** Schematic of the applications of an intelligent metasurface in everyday life. **b** Optical and EM images of the specimen, which include a single person with certain gestures. **c** Various hand gestures can be recognized by the intelligent metasurface system. **d** Monitoring of the breath rate when the EM wave is focused at the chest

The two works described above both (Fig. 3) involve a feedback system empowered by artificial neural networks or a microcontroller, and instructions are injected continuously into the dynamic metasurfaces to reconfigure their phase patterns in real time. The main difference between these approaches is that in ref. ^4^, the metasurface serves only as a radar to project the EM wave in desired directions or patterns based on the information collected from a separate sensor (e.g., gyroscope), while in ref. ^5^, the metasurface is involved in both the transmitting and receiving operations. As such, the two roles played by the same metasurface in ref. ^5^ require multiplexing in the time domain. These two pioneering studies represent a unique and powerful marriage between two fast-growing fields: metasurfaces and artificial intelligence, which will provide widespread applications in various fields.

**Fig. 3:**
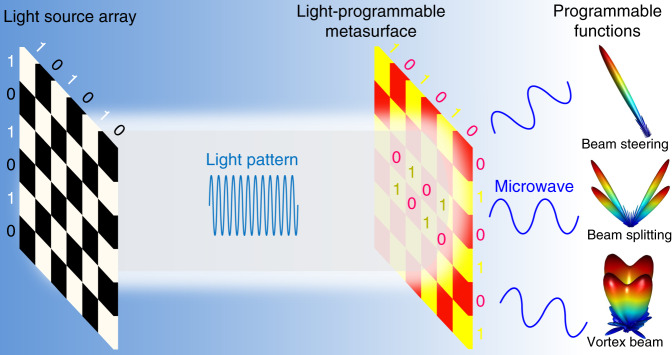
Schematic view of the operation of optically tuned dynamic metasurfaces. Patterned light from the left illuminates the EM metasurfaces with optically addressable individual pixels, resulting in dynamically tunable functionalities, including beam steering, beam splitting and vortex beam generation

Finally, in a more recent development, the authors show that electronic tuning of the metasurfaces in refs. ^2,4,5^ can be replaced by optical tuning, which leads to a number of advantages, including a reduction of the complexities of the circuit design^3^. In this approach, the metasurface design incorporates electronic varactors integrated with an optical interrogation network based on photodiodes. The phase distribution of the metasurface can be controlled dynamically by a patterned optical beam generated by an SLM. To demonstrate the effectiveness of this approach, the authors present a number of applications, including external cloaking, illusion, and dynamic vortex-beam generation, with very good performance. It is expected that the current approach can be incorporated with artificial intelligence in a similar manner as ref. ^4^ and ref. ^5^ with very slight modifications. More importantly, the implementation of optically controlled dynamic metasurfaces can be more readily extended to higher frequencies, such as terahertz and far infrared, than their electronically controlled counterparts, holding promise for wider applications.
